# Акромегалия при дифференциальной диагностике тугоухости

**DOI:** 10.14341/probl13249

**Published:** 2023-05-11

**Authors:** Л. К. Дзеранова, Л. И. Лепешкина, А. С. Шутова, М. А. Перепелова, Е. А. Пигарова, В. Н. Азизян, П. В. Акопян, Е. Г. Пржиялковская, Г. А. Мельниченко, Н. Г. Мокрышева

**Affiliations:** Национальный медицинский исследовательский центр эндокринологии; Российский университет дружбы народов; Национальный медицинский исследовательский центр эндокринологии; Национальный медицинский исследовательский центр эндокринологии; Национальный медицинский исследовательский центр эндокринологии; Национальный медицинский исследовательский центр эндокринологии; Национальный медицинский исследовательский центр эндокринологии; Национальный медицинский исследовательский центр эндокринологии; Национальный медицинский исследовательский центр эндокринологии; Национальный медицинский исследовательский центр эндокринологии

**Keywords:** акромегалия, тугоухость, соматотропный гормон, инсулиноподобный фактор роста 1, аденома гипофиза

## Abstract

Акромегалия — мультифакторное нейроэндокринное заболевание, обусловленное гиперпродукцией соматотропного гормона (СТГ). Более чем в 95% случаев акромегалия является следствием СТГ-секретирующей аденомы гипофиза. У пациентов с данным нейроэндокринным заболеванием медленно развивающийся симптомокомплекс может долгое время проявляться лишь сопутствующими патологическими состояниями, в том числе нарушениями слуховой функции в виде тугоухости. Трудности дифференциальной диагностики между осложнениями акромегалии и аналогичными нарушениями в виде самостоятельных нозологий на догоспитальном этапе обусловливают важность настороженности врачей смежных специальностей в отношении эндокринной патологии.

Нами представлен клинический случай улучшения слуховой функции на фоне комбинированного оперативного и консервативного лечения пациентки с макроаденомой гипофиза, акромегалией и тугоухостью.

Пациентка с активной стадией акромегалии и макроаденомой гипофиза размерами 57×35×32 мм с анте-, супра-, инфра-, параселлярным распространением (Knosp III(D), Knosp IV(S)) отмечала нарушение слуховой функции. Консультирована отоларингологом, диагностирована сенсоневральная тугоухость справа — 3-й степени, слева — 1-й степени. Пациентке проведено хирургическое лечение аденомы гипофиза, в раннем послеоперационном периоде отметила значительное улучшение слуховой функции. Через полгода проведена повторная аудиометрия, отмечен выраженный регресс поражений органов слуха.

Описанный нами случай указывает на обратимость редкого осложнения акромегалии — тугоухости и важность междисциплинарного подхода в ведении пациентов с данной патологией.

## Abstract (en)

Acromegaly is a multifactorial neuroendocrine disease caused by hyperproduction of growth hormone (GH). In more than 95% of cases the reason of acromegaly the GH-secreting pituitary adenoma. In patients with this neuroendocrine disease, a slowly developing complex of symptom can manifest with concomitant pathological conditions, including auditory function disordersDiagnostic difficulties of acromegaly at the ambulatory stage determine the importance of doctor`s awareness in different medical specialties.

Here we demonstrate a clinical case of the improvement of the auditory function due to combined surgical and medical treatment of a patient with the pituitary macroadenoma, acromegaly and hearing loss.

Anamnesis features: a patient with an active stage of acromegaly and a pituitary macroadenoma measuring 57x35x32 mm with ante-, supra-, infra-, parasellar spread, (Knosp III(D), Knosp IV(S) noted a violation of auditory function. She was consulted by an otolaryngologist, sensorineural hearing loss on the right of the 3rd degree was diagnosed, on the left of the 1st degree. The patient underwent surgical treatment of pituitary adenoma, noted a significant improvement in auditory function in the early postoperative period. Six months later, repeated audiometry was performed, marked regression of hearing damage was noted.

The case described by us indicates the reversibility of a rare complication of acromegaly — hearing loss and the importance of an interdisciplinary approach in the management of patients with this pathology.

## АКТУАЛЬНОСТЬ

Акромегалия представляет собой орфанную патологию, развивающуюся вследствие гиперпродукции соматотропного гормона (СТГ), чаще всего обусловленной наличием аденомы гипофиза. Рецепторы СТГ встречаются почти во всех тканях организма, вследствие чего клинические проявления его избытка наблюдаются со стороны различных органов и систем [1].

Наиболее частыми осложнениями акромегалии являются патология сердечно-сосудистой, дыхательной, костно-суставной систем и углеводного обмена, при этом данных, описывающих развитие нарушений слуха на фоне акромегалии, недостаточно [2]. Проявления аденомы могут мимикрировать под клиническую картину тугоухости. Известно, что поражения слухового аппарата могут проявляться в форме тугоухости по сенсоневральному, кондуктивному и смешанному типам. Информация о наличии слуховых нарушений вследствие акромегалии подтверждается некоторыми исследованиями, но их небольшое количество затрудняет оценку распространенности [3-5]. Патогенетически развитие тугоухости может быть обусловлено гипертрофией височной кости вследствие гиперпродукции гормона роста с последующим поражением слухового анализатора внутреннего уха. Сенсоневральный компонент, вероятнее всего, объясняется отеком слухового нерва и нарушением его микроциркуляции.

Нарушения слухового аппарата, в отличие от частых осложнений акромегалии, предположительно объясняются особенностями топографо-анатомического расположения опухоли, а также направлением и характером роста аденомы (распространением в носоглотку, сдавлением слухового нерва).

В настоящее время в соответствии с российскими клиническими рекомендациями по акромегалии аудиометрия и другие методы исследования слуха не являются обязательными этапами диагностики осложнений [6], в связи с чем нарушения работы слухового аппарата могут остаться незамеченными. Пациенты зачастую обращаются в первую очередь к специалистам смежных областей, в том числе, к врачам-оториноларингологам.

Данный клинический случай демонстрирует важность междисциплинарного подхода в дифференциальной диагностике и лечении тугоухости при акромегалии.

## ОПИСАНИЕ СЛУЧАЯ

Пациентка А., 29 лет, впервые обратилась в ФГБУ «НМИЦ эндокринологии» Минздрава России в марте 2022 г. с жалобами на головную боль, общую слабость, укрупнение черт лица, увеличение пальцев рук и увеличение размера ноги, снижение слуха и остроты зрения, затрудненное носовое дыхание, шум в голове преимущественно в ночное время, запоры, храп и боль в коленных суставах.

Из анамнеза известно, что пациентка считает себя больной с 2018 г., когда начали беспокоить нарушение менструального цикла, повышенная потливость и угревая сыпь. Тогда же пациентка обратилась к гинекологу, был назначен комбинированный гормональный контрацептивный препарат, содержащий дроспиренон 30 мг+ этинилэстрадиол 0,02 мг + кальция левомефолат 0,451 мг, на фоне приема которого отмечена нормализация цикла, однако при отмене препарата регулярность менструаций вновь нарушилась.

В 2020 г. появились головные боли, однако за медицинской помощью не обращалась. В 2021 г. начала отмечать изменения черт лица, увеличение размеров кистей рук и стоп, снижение зрения и ухудшение слуха, в связи с чем проведено обследование по месту жительства. Выявлено повышение уровня СТГ в сыворотке крови до 200 нг/мл, пролактина (ПРЛ) до 1171 мкМЕ/мл (норма до 496). Отмечено нарушение углеводного обмена: гликемия натощак 6,9 ммоль/л (уровень гликированного гемоглобина HbA1c — 6,6%), что также характерно для акромегалии.

Выполнена магнитно-резонансная томография (МРТ) головного мозга, которая позволила визуализировать кистозно-солидное образование — аденому гипофиза больших размеров, 57×35×32 мм, локализованную в полости турецкого седла и распространяющуюся в область супраселлярной цистерны, в пазуху клиновидной кости и кавернозные синусы (рис. 1). Кроме этого, отмечена инвазия образования в область верхушки правой орбиты, задние ячейки решетчатой кости справа и носоглотку, с сужением ее просвета на одну треть. Аденома смещала и компримировала перекрест зрительных нервов, воронка и задняя доля гипофиза не дифференцировались. Степень инвазии аденомы соответствовала III(D), IV(S) степени в соответствии со шкалой Knosp.

Для уточнения диагноза и определения тактики лечения пациентка направлена в ФГБУ «НМИЦ эндокринологии» Минздрава России. Учитывая характер жалоб и фенотипические признаки акромегалии, проведено повторное определение уровня инсулиноподобного фактора роста 1 (ИФР-1) — 645,2 нг/мл (до 311) и СТГ более 80 нг/мл (до 6,9), что подтвердило активную стадию акромегалии. Также выявлено повышение уровня ПРЛ до 1086 мЕд/л при верхней границе референсных значений до 500.

В связи с жалобами на заложенность носа и снижение слуха был проведен осмотр врачом-оториноларингологом. По результатам риноскопии отмечено сужение носовых ходов за счет искривления носовой перегородки и отечности носовых раковин с обеих сторон. Также на основании проведенной аудиометрии (рис. 1) диагностирована хроническая двусторонняя сенсоневральная тугоухость справа — III степени, слева — I степени.

По итогам проведенных исследований установлен диагноз: Акромегалия, активная стадия; макроаденома гипофиза с анте-, супра-, инфра-, параселлярным распространением. Уточнить генез выявленной у пациентки гиперпролактинемии на момент исследования не представлялось возможным, поскольку она могла быть обусловлена смешанной гормональной активностью аденомы, stalk-эффектом вследствие компрессии аденомой воронки гипофиза или сочетанием обеих причин.

Пациентка консультирована нейрохирургом, принято решение о необходимости проведения оперативного лечения в объеме трансназальной аденомэктомии.

При удалении аденома представляла собой образование беловато-серого цвета, мягкой консистенции с небольшим кистозным компонентом, занимавшее всю полость турецкого седла, распространявшееся в инфра- и супраселлярном направлении и прораставшее медиальную стенку левого кавернозного синуса с множественными участками поверхностной инвазии твердой мозговой оболочки в области турецкого седла.

При последующем проведении иммуногистохимического исследования ткани опухоли использованы панели антител к СТГ, ПРЛ, низкомолекулярному цитокератину САМ 5.2, соматостатиновым рецепторам 2-го и 5-го типа. Выявлена умеренно выраженная экспрессия рецепторов соматостатина 2-го подтипа на мембране до 80% опухолевых клеток (6 баллов по IRS), умеренно выраженная экспрессия рецепторов соматостатина 5-го подтипа на мембране более 80% опухолевых клеток (8 баллов по IRS), экспрессия ПРЛ — в единичных опухолевых клетках. Клиническая картина и результаты иммуногистохимического анализа указывают на наличие редкогранулированной соматотрофной опухоли, характеризующейся инвазивным ростом и изначальной резистентностью к аналогам соматостатина 1-й генерации.Повышение уровня ПРЛ связано не с наличием лактотрофного компонента, а со сдавлением ножки гипофиза.

В послеоперационном периоде данных за вторичный гипотиреоз, несахарный диабет, вторичную надпочечниковую недостаточность получено не было, выявлен вторичный гипогонадизм. При этом пациентка отчетливо отметила улучшение слуха уже в первый день после операции. Уровень СТГ в ходе проведенного перорального глюкозотолерантного теста через 7 дней после операции остался не подавленным, вследствие чего диагностировано отсутствие ремиссии акромегалии, инициирована медикаментозная терапия аналогами соматостатина длительного действия (Октреотид Лонг 20 мг 1 раз в 28 дней), агонистами дофамина (Каберголин 0,5 мг 1 раз в неделю).

Через полгода после оперативного лечения пациентке проведена повторная аудиометрия (рис. 3), по данным которой подтверждена положительная динамика относительно улучшения слуховой функции: установлен диагноз двусторонней хронической сенсоневральной тугоухости I степени.

По данным лабораторных исследований уровень ПРЛ на терапии агонистами дофамина — 182,5 мЕд/л (94–500), коррекции дозы не потребовалось. Также диагностирована резистентность к аналогам соматостатина — ИФР-1 634,1 нг/мл (78–311), инициирована терапия препаратом из класса антагонистов рецептора СТГ — пэгвисомантом 15 мг подкожно ежедневно. При контроле в динамике через 3 дня наблюдались хорошая переносимость препарата, улучшение общего самочувствия и слуховой функции.

**Figure:**
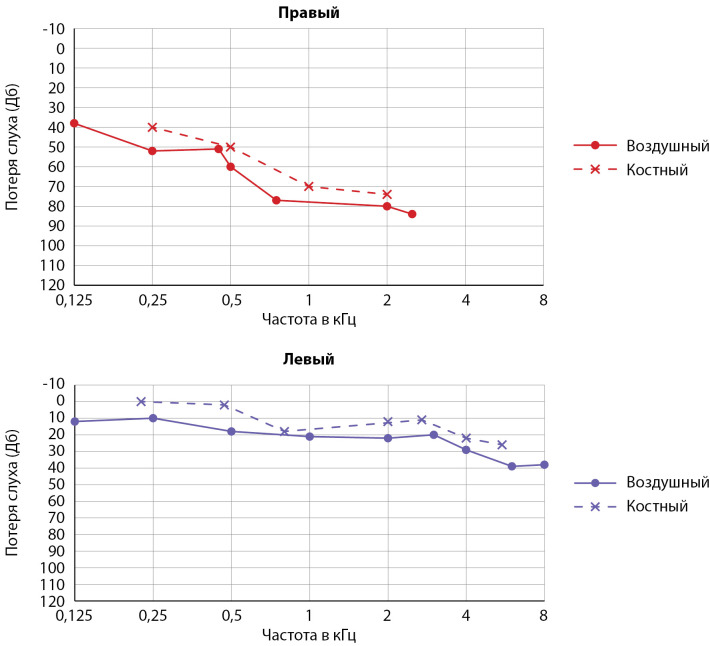
Рисунок 1. Аудиограмма до операции. Сенсоневральная тугоухость справа — III степени, слева — I степени. Дб — децибел, кГц — килогерц.

**Figure:**
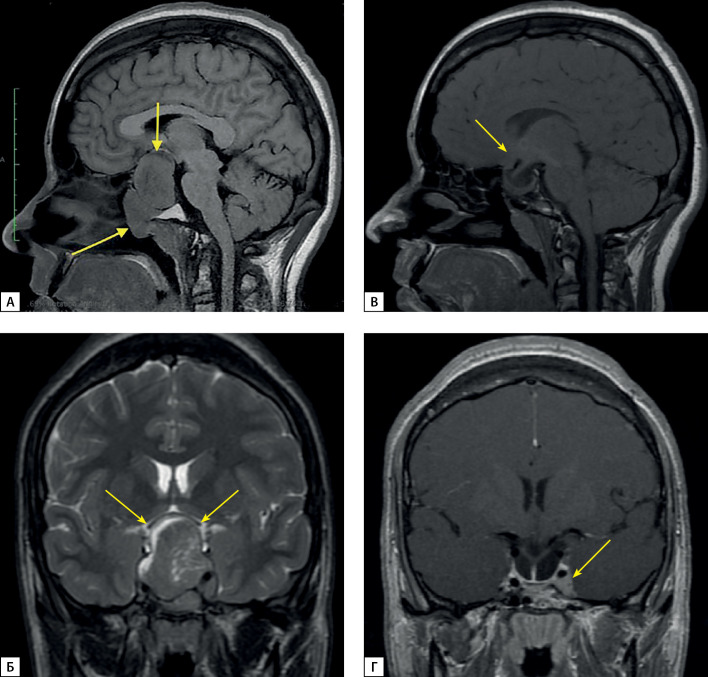
Рисунок 2. МРТ головного мозга с внутривенным контрастированием А, Б — макроаденома гипофиза до операции. Сагиттальная и фронтальная проекции соответственно; В, Г — Послеоперационные изменения, остаточная ткань аденомы. Сагиттальная и фронтальная проекции соответственно.

**Figure:**
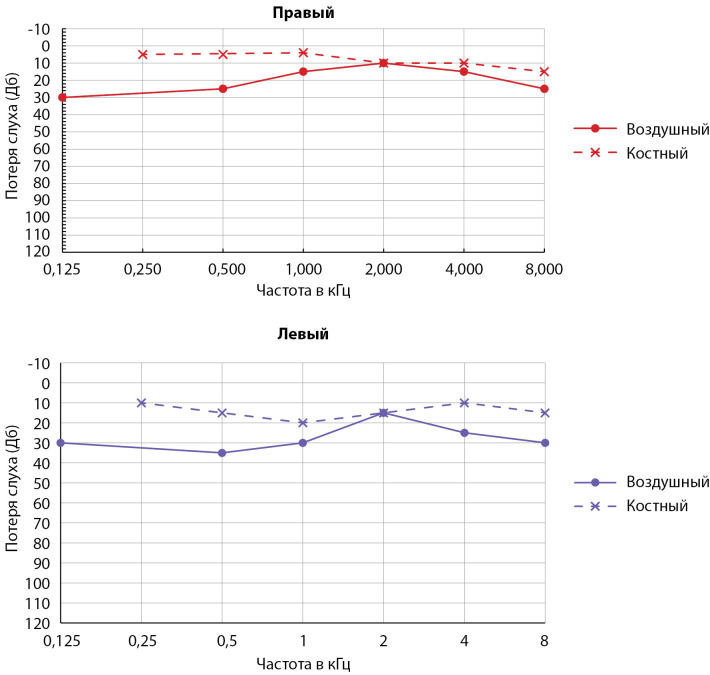
Рисунок 3. Аудиограмма после операции. Двусторонняя сенсоневральная тугоухость I степени. Дб — децибел, кГц — килогерц.

## ОБСУЖДЕНИЕ

У пациентов с акромегалией тугоухость может носить различный характер. При поражении структур среднего уха или барабанной перепонки развивается картина кондуктивной тугоухости, при нарушениях во внутреннем ухе или в улитке — сенсоневральной [7]. Развитие тугоухости по сенсоневральному типу предполагаемо, по данным источников литературы, происходит вследствие гипертрофии височной кости и последующего сужения внутреннего слухового прохода [3]. В литературе встречаются описания случаев инфраселлярной инвазии аденомы гипофиза в носоглотку с последующей дисфункцией слуховых труб и нарушением вентиляции барабанной полости [5]. Также развитие сенсоневральной тугоухости у пациентов с акромегалией может быть обусловлено поражением вышележащих структур, в том числе ствола и подкорковых центров головного мозга, являющихся частью слухового анализатора. W. Pilecki и соавт. отметили, что снижение миелинизации слуховых путей ствола мозга, вероятно, возникает из-за изменений в метаболизме глюкозы, что также характерно для пациентов с акромегалией и может вызывать поражение слухового аппарата [8]. E.C. Kuan и соавт. провели исследование пациентов с акромегалией после хирургического лечения и указывают, что механизм возникновения сенсоневральной тугоухости неизвестен [9].

Возникновение кондуктивной тугоухости объясняется отосклерозом и гипертрофией слуховых косточек, возможно, в результате действия СТГ [4].

В исследовании 2016 г. S. Tabur и соавт. проводилось обследование органов слуха у 41 пациентов с акромегалией, среди которых у 13 (32%) выявлена тугоухость, из них у 54% — по сенсоневральному, у 46% — кондуктивному типу [5]. У пациентов с акромегалией показатели аудиометрии были хуже, чем в контрольной группе, выявлена связь нарушений слуховой функции с высокими уровнями СТГ и ИФР-1. Разрастание мягких тканей и костных структур в эпифаринксе и евстахиевой трубе рассматривается как причина обструкции евстахиевой трубы, приводящей к дисфункции среднего уха при акромегалии и в конечном итоге — тугоухости. Также авторы отмечают нарушение сокращения стременной мышцы, вероятно, за счет отосклероза, что может быть также связано с поражением черепно-мозговых нервов и центральных структур головного мозга на фоне снижения миелинизации нервов из-за метаболических нарушений. [5].

K. Audin и соавт. провели исследование 43 пациентов с диагностированной акромегалией, где на основании результатов аудиометрии и КТ были выявлены случаи кондуктивной (9%), сенсоневральной (30%) и смешанной (18%) тугоухости [10]. Вероятными причинами появления данной симптоматики являются нарушенная вентиляция среднего уха, сужение внутреннего слухового прохода, фиксация головки молоточка и изменения плотности ткани слуховых косточек. В данном клиническом случае улучшение слуховой функции, вероятнее всего, обусловлено уменьшением отека слухового нерва после удаления основного объема опухоли. Также отмечалось значимое снижение уровня СТГ (до операции — более 80 нг/мл, после операции — 11,1 нг/мл).

Основные исследования направлены на изучение распространенности тугоухости у пациентов с акромегалией, а не на причины возникновения, что обусловлено редкостью этого нейроэндокринного заболевания. Разнородность групп, малая выборка не дают полноценно составить картину патогенеза тугоухости [11]. Учитывая высокую вероятность нарушения органов слуха у пациентов с акромегалией, необходимо продолжить исследования поражения слухового аппарата при данной патологии, цель которых — установление особенностей патогенеза нарушения слуха при акромегалии и изучение зависимости степени выраженности поражения от течения основного заболевания и обратимости на фоне лечения. Полученные результаты позволят своевременно выявлять и корректировать имеющиеся нарушения, а также будут способствовать улучшению алгоритма помощи пациентам с акромегалией.

## ЗАКЛЮЧЕНИЕ

Описанный нами клинический случай подтверждает, что врачи смежных специальностей, в особенности врачи-отоларингологи, должны быть осведомлены о разнообразии симптомов и осложнений акромегалии, обращать внимание на наличие типичных проявлений: увеличение дистальных отделов конечностей, грубые черты лица и кожные складки, повышенную потливость, акне, гирсутизм, ночное апноэ и своевременно направлять пациента к врачу-эндокринологу.

Клинические проявления акромегалии поражают своим многообразием и могут затрагивать сердечно-сосудистую, дыхательную, опорно-двигательную системы и даже слуховой аппарат. После нейрохирургического или медикаментозного лечения может наблюдаться регресс некоторых симптомов и осложнений акромегалии, например, головной и суставной боли [12], нарушений углеводного, липидного обменов [13] и патологии слухового аппарата.

Гетерогенность клинической картины акромегалии, возможная обратимость осложнений на фоне лечения имеют ключевое значение в определении персонализированной тактики ведения пациента.

## ДОПОЛНИТЕЛЬНАЯ ИНФОРМАЦИЯ

Источники финансирования. Работа выполнена по инициативе авторов без привлечения финансирования.

Конфликт интересов. Авторы декларируют отсутствие явных и потенциальных конфликтов интересов, связанных с содержанием настоящей статьи.

Участие авторов. Дзеранова Л.К. — получение, анализ данных, внесение в рукопись важной правки; Лепешкина Л.И. — получение, анализ данных, написание статьи; Шутова А.С. — получение, анализ данных, написание статьи; Перепелова М.А. — получение, анализ данных, написание статьи; Пигарова Е.А. — получение, анализ данных, внесение в рукопись важной правки; Азизян В.Н. — проведение хирургического лечения аденомы гипофиза, внесение в рукопись важной правки; Акопян П.В. — проведение аудиометрии, внесение в рукопись важной правки; Пржиялковская Е.Г. — получение, анализ данных, внесение в рукопись важной правки; Мельниченко Г.А. — внесение в рукопись важной правки, одобрение финальной версии; Мокрышева Н.Г. — внесение в рукопись важной правки, одобрение финальной версии.

Все авторы одобрили финальную версию статьи перед публикацией, выразили согласие нести ответственность за все аспекты работы, подразумевающую надлежащее изучение и решение вопросов, связанных с точностью или добросовестностью любой части работы.

Согласие пациента. Пациентка добровольно подписала информированное согласие на публикацию персональной медицинской информации в обезличенной форме в журнале «Проблемы эндокринологии».
